# Multiple-clone infections of *Plasmodium vivax*: definition of a panel of markers for molecular epidemiology

**DOI:** 10.1186/s12936-015-0846-5

**Published:** 2015-08-25

**Authors:** Aracele M. de Souza, Flávia C. F. de Araújo, Cor J. F. Fontes, Luzia H. Carvalho, Cristiana F. A. de Brito, Taís N. de Sousa

**Affiliations:** Centro de Pesquisas René Rachou, Fundação Oswaldo Cruz (FIOCRUZ), Belo Horizonte, Minas Gerais Brazil; Hospital Julio Muller, Universidade Federal de Mato Grosso, Cuiabá, Mato Grosso Brazil

**Keywords:** Malaria, *Plasmodium vivax*, Multiple-clone infection, Molecular markers, Genetic variability, PCR-capillary electrophoresis-based method, Molecular epidemiology

## Abstract

**Background:**

*Plasmodium vivax* infections commonly contain multiple genetically distinct parasite clones. The detection of multiple-clone infections depends on several factors, such as the accuracy of the genotyping method, and the type and number of the molecular markers analysed. Characterizing the multiplicity of infection has broad implications that range from population genetic studies of the parasite to malaria treatment and control. This study compared and evaluated the efficiency of neutral and non-neutral markers that are widely used in studies of molecular epidemiology to detect the multiplicity of *P. vivax* infection.

**Methods:**

The performance of six markers was evaluated using 11 mixtures of DNA with well-defined proportions of two different parasite genotypes for each marker. These mixtures were generated by mixing cloned PCR products or patient-derived genomic DNA. In addition, 51 samples of natural infections from the Brazil were genotyped for all markers. The PCR-capillary electrophoresis-based method was used to permit direct comparisons among the markers. The criteria for differentiating minor peaks from artifacts were also evaluated.

**Results:**

The analysis of DNA mixtures showed that the tandem repeat *MN21* and the polymorphic blocks 2 (*msp1B2*) and 10 (*msp1B10*) of *merozoite surface protein*-*1* allowed for the estimation of the expected ratio of both alleles in the majority of preparations. Nevertheless, *msp1B2* was not able to detect the majority of multiple-clone infections in field samples; it identified only 6 % of these infections. The *merozoite surface protein*-*3 alpha* and microsatellites (*PvMS6* and *PvMS7*) did not accurately estimate the relative clonal proportions in artificial mixtures, but the microsatellites performed well in detecting natural multiple-clone infections. Notably, the use of a less stringent criterion to score rare alleles significantly increased the sensitivity of the detection of multi-clonal infections.

**Conclusions:**

Depending on the type of marker used, a considerable amplification bias was observed, which may have serious implications for the characterization of the complexity of a *P. vivax* infection. Based on the performance of markers in artificial mixtures of DNA and natural infections, a minimum panel of four genetic markers (*PvMS6*, *PvMS7*, *MN21*, and *msp1B10*) was defined, and these markers are highly informative regarding the genetic variability of *P. vivax* populations.

**Electronic supplementary material:**

The online version of this article (doi:10.1186/s12936-015-0846-5) contains supplementary material, which is available to authorized users.

## Background

*Plasmodium vivax* is globally the most widely distributed *Plasmodium* species that infects humans, being common in tropical and sub-tropical areas outside of Africa [1, 2]. Several factors have highlighted the clinical importance of malaria caused by *P. vivax*, such as the spread of parasite resistance to available drugs [3]. In addition, the concept of vivax malaria as a benign disease has evolved with the description of severe cases and even deaths [4–6]. Finally, dormant forms of the parasite in the liver, i.e., hypnozoites, act as a reservoir for the disease and have hindered the control of malaria caused by *P. vivax* [7]. These factors have all increased interest in vivax malaria, primarily in the new Malaria Eradication Research Agenda (malEra) [8].

*Plasmodium vivax* infections are often characterized by the presence of two or more genetically distinct parasites in the same individual [9–11]. These infections are very common in malaria-endemic areas worldwide [12–11] and can arise from a single mosquito bite carrying a mixture of parasites or from inoculation by different mosquitoes carrying single clones. Additionally, relapses of *P. vivax* infection due the reactivation of hypnozoites can contribute to increased clonal diversity. As a result, the association between the multiplicity of *P. vivax* infection and malaria endemicity is weak, with areas of low endemicity sometimes featuring high rates of multiple infections [10–12]. The number of parasite clones in a patient varies greatly, and some infections contain up to nine clones [12]. Characterizing the multiplicity of infection has broad implications ranging from population genetic studies of the parasite to malaria treatment and control. First, evolutionary and population genetic studies rely on accurate parasite genotype/haplotype inference, which is non-trivial when more than one clone is present and clones differ at examined loci [13, 14]. Second, characterizing the within-host diversity is essential to address several issues, such as differentiation between new infection and recrudescence, in order to better estimate the true risk of treatment failure and explore the dynamics of clones influenced by host immunity during anti-malarial treatment or challenge with vaccine [12, 15, 16]. Third, malaria patients infected by multiple parasite strains have been shown to be at a higher risk of treatment failure [17]. Thus, a broad understanding of the genetic diversity of parasite populations can contribute to the definition of control measures, including an appropriate anti-malarial treatment.

The publication of the complete genome sequence of *P. vivax* has led to the discovery of many molecular markers, such as microsatellites, tandem repeats and single nucleotide polymorphisms (SNPs) [18]. These markers have proven useful for population genetic studies and for the characterization of the multiplicity of *P. vivax* infections. However, many studies have shown that the characterization of multi-clonal infections depends on both the accuracy of the genotyping method, and the type and number of the molecular markers analysed [19, 20]. Thus, the use of different approaches may significantly affect the ability to detect multi-clonal *P. vivax* infections and may hinder comparability among studies [21, 22]. Furthermore, the method used may influence the estimation of the relative abundance of clones in multiple infections.

This study evaluated and compared the ability of different molecular markers—two microsatellites, one tandem repeat and three antigen-coding genes—to estimate the number and the relative abundance of alleles present in multi-clonal *P. vivax* infections. In order to simulate multiple-clone infections with well-defined proportions of different parasite genotypes, cloned PCR products or patient-derived genomic DNA were artificially mixed. In addition, the performance of these markers was also evaluated by genotyping *P. vivax* isolates that had infected patients from the Brazilian Amazon. The PCR-capillary electrophoresis-based method (PCR-CE), which offers several advantages, such as high resolution (1 bp), reproducibility in determining fragment size, and a cost-benefit for the analysis of a large number of field samples, was used to genotype all markers [23]. Although this method of quantification is subject to some limitations, many studies have shown that the peak heights correspond to the actual relative proportions of clones in an infection when data are properly normalized [19, 24, 25]. The ability to identify less abundant clones depends on the criteria applied to differentiate minor peak from artifacts, which allows the multiplicity of infection to be properly defined. Two criteria are commonly used to score multiple alleles per locus: cut-off values for minor peak detection of (1) one-fourth or (2) one-third the height of the predominant peak [9, 12, 26–29]. Although the one-third criterion is the most widely used, few studies have evaluated the sensitivity of these criteria for the detection of multi-clonal infections [19]. Here, the results showed the necessity to apply the less stringent one-fourth criterion to increase the detection of multiple-clone infections. Specifically, a minimum panel of four markers was defined to characterize the multiplicity of a *P. vivax* infection.

This is the first study to show that depending on the type of marker used for *P. vivax* analysis, a considerable amplification bias is observed. This relationship may have serious implications for the characterization of the complexity of an infection. Moreover, these findings were facilitated by the use of parasite DNA samples with well-defined proportions of each genotype in artificial mixtures as well as the use of molecular markers with different features, such as neutral and non-neutral markers.

## Methods

### Field isolates and DNA extraction

Fifty-one *P. vivax* isolates were obtained from the blood of infected patients from the Brazilian Amazon, including 22 samples previously characterized as having multi-clonal infection [9, 11]. The mono-infections were confirmed by 6 to 11 molecular markers described here or elsewhere [9, 11]. DNA was extracted from whole blood samples using the Gentra Puregene Blood Kit (Qiagen, Valencia, CA, USA), according to the manufacturer’s protocol. Ethical approval for the study was obtained from the Ethics and Research Committee of René Rachou Research Center (Protocol number 20/2009).

### Artificial mixtures of DNA

To simulate multiple-clone infections with a known proportion of each clone, two plasmid DNAs (pDNA), each containing distinct variants of each locus (A-type and B-type alleles), were mixed in different proportions to produce 11 different mixtures prior to PCR amplification and genotyping. To set up mixtures, each pDNA was quantified in a NanoDrop 2000 spectrophotometer (Thermo Scientific, San Jose, CA, USA), and the number of plasmid copies was estimated as follows: plasmid copy number = [(6.02 × 10^23^ copies/mol) × DNA quantity (g)]/[DNA length (bp) × 660 (g/mol/bp)]. Two-fold serial dilutions of pDNA were prepared for each experiment, with DNA concentrations ranging from 3.13 × 10^7^ to 1.0 × 10^9^ copies/µL of the plasmid. For all PCR reactions, 1 µL of each plasmid in a specific concentration was added to reaction to produce a curve with varied proportions of pDNA. In addition, mixed genomic DNA (gDNA) samples from two *P. vivax*-infected patients previously identified as being mono-clonal were also used to simulate multi-clonal infections [9]. To this end, samples with similar parasitaemia (microscopy) and leukocyte counts (leukogram determination) were selected, and the gDNA concentration was determined using a spectrophotometer. The parasite density in samples from *P. vivax*-infected patients ranged from 3000 to 7500 parasites/µL, as determined by light microscopy. The genomic DNA was two-fold serially diluted (8–0.25 ng), and 1 µL of each sample was added to each PCR reaction. To estimate the relative abundance of alleles in the mixture after genotyping, the ratio between the two peak heights (given in arbitrary fluorescence units) was calculated for each mixture. Data shown represent the average of two independent experiments, and each experiment was analysed in duplicate in an automatic DNA sequencer.

The observed data were normalized according to Havryliuk et al. [17] to allow direct comparisons between expected ratios and observed values. Specifically, the peak height ratios observed in a 1:1 mixture were used as normalization factors in subsequent analyses.

### PCR amplification and genotyping of molecular markers

The six loci selected for this study consisted of two microsatellites (*PvMS6* and *PvMS7*), one tandem repeat (*MN21*), and blocks 2 and 10 of *merozoite surface protein*-*1* (*msp1B2* and *msp1B10*) and *merozoite surface protein*-*3 alpha* (*msp3α*). These fragments were amplified using specific primers and conditions as described previously [10, 11, 34]. The nested approach described by Koepfli et al. [10] was modified to capture more variability and used to amplify block 2 of *msp1* and *msp3α*. The primers used in the nested reaction were changed to the following: *msp1B2* (forward primer 5′GACGATATTGGAAAATTGGA3′; reverse primer 5′CTCCTTCAGCACTTTCACGCGCTT3′); and for *msp3α* (forward primer 5′CCCGCATGAGGAGCCAAACAACTT3′; reverse primer 5′CCTTTGCATTTTTTGCCGCAG3′). For *msp1b2*, these new primers anneal in a more conserved region of the gene, and the new *msp3α* primers were designed to amplify a larger and more polymorphic fragment. All forward primers used in the genotyping were conjugated to the fluorescent dye 6-FAM. The following mixture was subjected to nested PCR: 1 μL of PCR product of the first reaction (diluted 1:1000 in H_2_O), 1 µM of each primer (Integrated DNA Technologies, San Diego, CA, USA), 0.125 mM of dNTPs, 2 μL of 10X *Taq* polymerase buffer (Invitrogen, Carlsbad, CA, USA) and 1 U of recombinant *Taq* polymerase (Invitrogen) in a final volume of 20 μL. The magnesium concentration varied from 3 mM (*msp1B2*) to 2 mM (*msp3α*). The cycling parameters were set to the following: 1 cycle at 94 °C for 4 min; 25 cycles consisting of 40 s of denaturation at 94 °C, 30 s of annealing at 64 °C (for *msp1B2*) or 63 °C (*msp3α*), and 40 s of elongation at 72 °C; and a final cycle of 10 min at 72 °C.

For capillary electrophoresis, 2 μL of the diluted PCR products (1:20 in H_2_O) was mixed with the size standard ET-Rox 550 or ET-Rox 900 (Amersham Biosciences) diluted 1:40 in 0.1 % Tween-20 to a final volume of 10 μL. After capillary electrophoresis, alleles were visualized and scored in an automatic DNA sequencer (MegaBACE; Amersham Biosciences, Piscataway, NJ, USA). Their lengths and relative abundance (peak heights in electropherograms) were determined using the MegaBACE™ Fragment Profiler version 1.2 software (Amersham Biosciences). For the electropherogram analysis, the minimum peak height was set to 150 arbitrary fluorescence units (rFU). Additionally, cut-off values for minor peak detection of one-fourth or one-third the height of the predominant peak were used to exclude artifact peaks.

### Cloning of PCR products

For each locus, two variants differing in size were cloned into the pGEM-T Vector (Promega) or TOPO-TA Cloning Vector (Invitrogen): 305 or 203 bp for *PvMS6*; 362 or 350 bp for *PvMS7*; 641 or 692 bp for *msp3α*; 402 or 429 bp for *msp1B2*; 252 or 258 bp for *msp1B10*; 292 or 257 bp for *MN21* (Additional file 1). The fragments cloned into the pGEM-T Vector were termed A-type, and those cloned into the TOPO-TA Cloning Vector were termed B-type. The PCR amplicons were cloned according to the manufacturer’s protocol. Recombinant vectors were used to transform the competent *Escherichia coli* Top10 strain using thermal shock, and the cells were plated in LB agar supplemented with ampicillin (50 μg/mL), X-Gal (1 mg/mL) and IPTG (0.1 mM) [30]. The plasmid DNAs were extracted using a QIAprep Spin Miniprep Kit (Qiagen).

## Results

### Evaluation of amplification bias in artificial mixtures of plasmid DNA

Mixtures of DNA were prepared using known proportions of two different pDNAs (cloning vectors, each containing one of the two variants of a locus studied). Overall, the efficiencies of six genetic markers (two microsatellites, one tandem repeat, and three antigen-coding markers) to detect alleles in artificial mixtures were compared. All six molecular markers accurately identified the correct alleles in samples containing a single clone. However, an amplification bias was detected for most markers in the 1:1 mixtures; specifically, one of the two alleles at a given locus was predominantly amplified (Additional files 2 and 3).

**Fig. 1:**
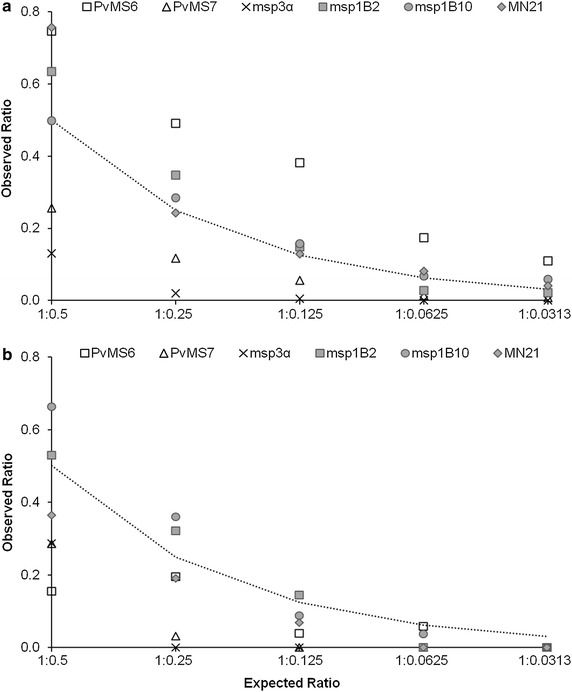
Expected and normalized observed ratios of molecular marker alleles amplified from mixtures of cloned DNA with predominance of A-type allele (**a**) and B-type allele (**b**). Two microsatellite markers (*PvMs6* and *PvMS7*), one tandem repeat (*MN21*), and three antigen-coding markers (*msp1B2*, *msp1B10* and *msp3α*) were PCR amplified and analysed by capillary electrophoresis. The *dotted line* indicates the expected ratios of peak heights according to the proportion of molecules (cloned DNA) from each allele used as a template for PCR amplification. Data represent the average of two independent experiments

Only three of the six markers (*msp1B2*, *msp1B10* and the tandem repeat *MN21*) were able to detect the correct proportions of each clone in most of the artificial mixtures (Fig. 1 and Additional file 2). For both *msp1* markers, the correspondence between the expected and observed proportions was evident, even for non-normalized data (Additional file 2). Thus, six to seven of ten tested dilutions corresponded to the expected proportions of the two cloned *msp1* markers in the infection (Fisher´s exact test, *P* > 0.05). These results agree with linear regression analyses, which indicated a significant relationship between the actual peak height and the known mixture of pDNA based on high regression values (*R*^*2*^) and a slope close to the expected value of unity for both *msp1* markers and *MN21* (Additional file 2).

In most mixtures, the correct proportions of the two clones were not accurately determined using the microsatellite markers *PvMS6* and *PvMS7* (Additional file 2). Nevertheless, these markers were sensitive to the decreased relative abundance of alleles in artificial mixtures, mainly when the A-type allele was predominant (for mixtures ranging from 1:0.5 to 1:0.0313) (Fig. 1a). For *PvMS6*, the analysis of the non-normalized data showed that the shorter (B-type) allele was preferentially amplified, even when the expected ratio of A-type to B-type alleles was as high as 1:0.125 (Additional file 2). For example, the B-type allele clearly predominated (1:1.49) for this expected ratio (1:0.125). A similar result was observed for mixtures of genomic DNA without cloning (Additional file 4).

### Criteria for detection of rare alleles in artificial mixtures of plasmid DNA

**Fig. 2:**
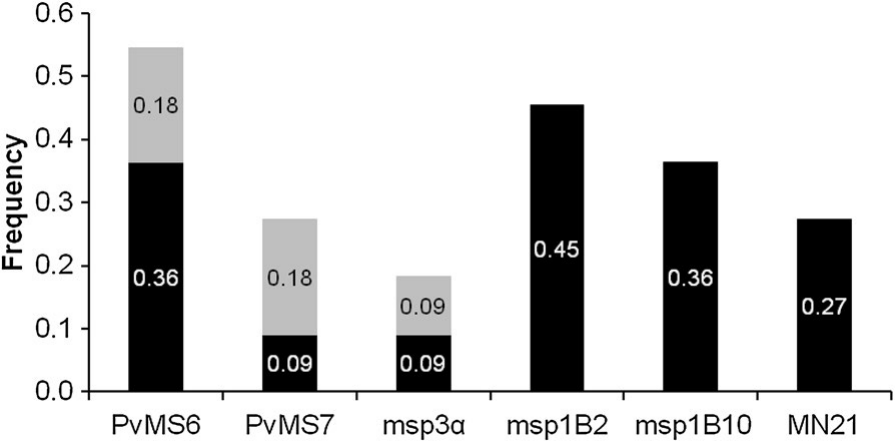
Detection of alleles in artificial mixtures of plasmid DNA by applying different criteria for rare allele identification. The frequency of detection of multiple alleles was calculated considering all 11 of the artificial mixtures assayed for each marker. The bars represent the total proportion of infections identified by each marker. Two criteria for minor allele detection were considered: a cut-off value of one-third (colored in *black* only) or one-fourth (the entire *bar*, including both *black* and *grey* *bars*), of the height of the predominant peak. The increase in the rate of detection of alleles with one-fourth criterion is highlighted in *grey*

Two criteria are frequently used to score rare alleles in multiple-clone infections: (1) the one-third criterion, wherein peak heights of rare alleles are equal or higher than 33 % of the height of the predominant peak, and (2) the one-fourth criterion, in which a cut-off value of 25 % is applied to detect rare alleles. When the one-third criterion was applied, *PvMS6* and both *msp1* markers detected 36–45 % of multiple infections (Fig. 2). For *PvMS6*, the use of the one-fourth criterion allowed an increase of 18 % in the detection rate of multiple infections, increasing the overall rate to 54 %. For *PvMS7*, *msp3α* and *MN21*, the multi-clonal parasite rates were below 30 % using either of the cut-off value criteria (25 or 33 %).

Based on the tested mixtures, the application of the one-third criterion allowed the detection of the rare alleles up to a dilution of 1:0.0625 (16× diluted), whereas the one-fourth criterion permitted the identification of rare alleles in more unbalanced mixtures (32x diluted) (Additional file 5). Overall, the use of the one-fourth criterion improved the detection rate of multiple infections up to 33 %, as shown for 1:1 mixtures.

### Genotyping of *Plasmodium vivax* isolates from natural infections

**Fig. 3:**
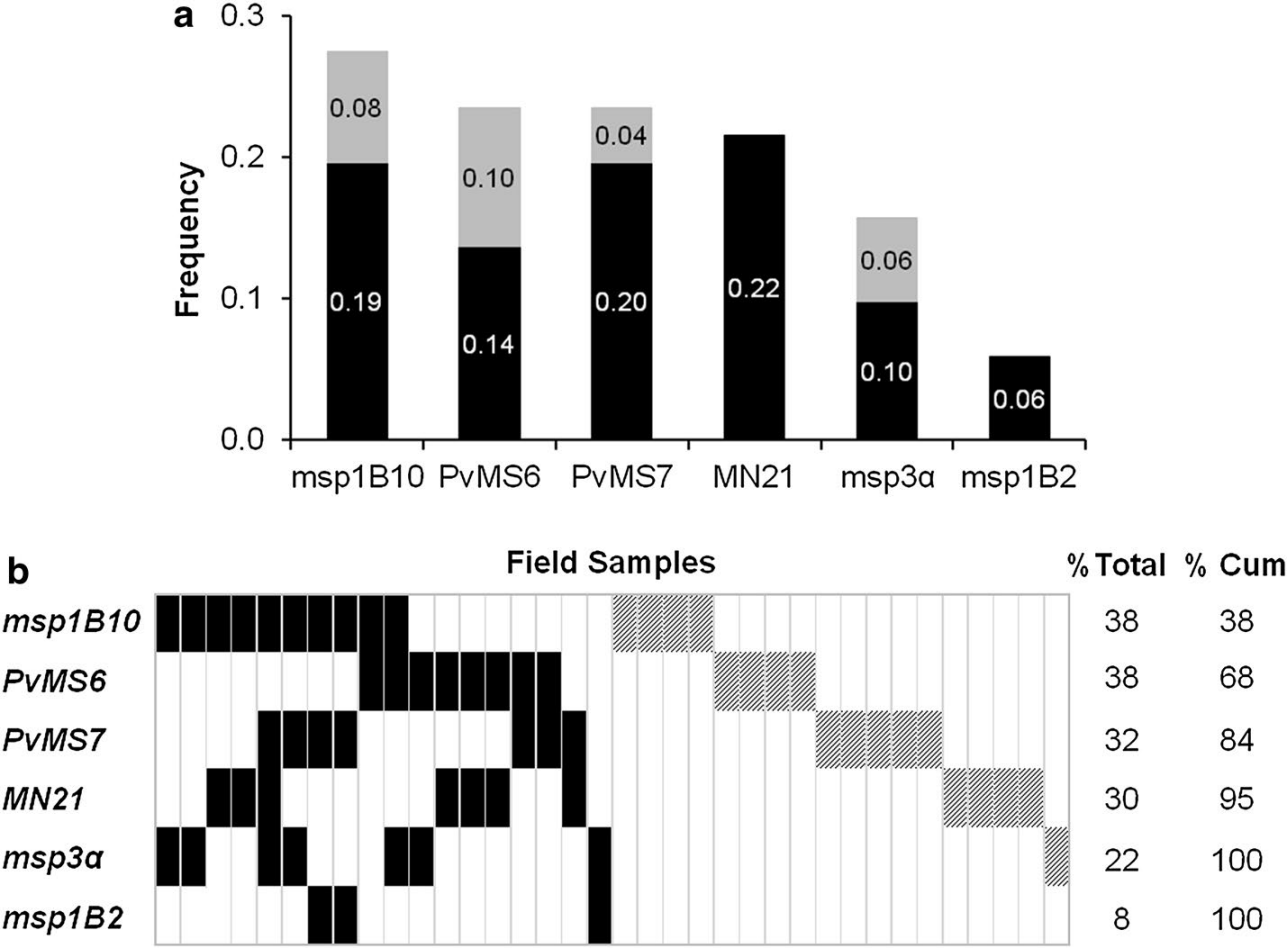
Detection of multiple-clone infections in field samples. The six markers were genotyped in 51 samples from malaria-endemic area of Brazil. A cut-off value of one-fourth was considered for the analysis. **a** The frequency of multi-clonal infections was calculated considering both criteria for minor allele detection: a cut-off value of one-third (*black bars* only) or one-fourth (the entire *bar*, including both *black* and *grey bars*) of the height of the predominant peak. The increase in the rate of detection of multiple-clone infections with the one-fourth criterion is highlighted in grey. **b** Results of genotyping are shown for the 36 samples with multiple-clone infection detected by one (*hatched rectangles*) or more markers (*rectangles* colored in *black*). Each column represents the same sample genotyped with the six markers. The frequency of multi-clonal infections (% Total) was calculated for each marker. The increase in the number of multiple-clone infections detected, resulting from the combination of two or more markers, is indicated as the cumulative percentage (% Cum)

Fifty one samples from the Brazilian Amazon were genotyped with the six markers based on the one-fourth criterion, and 36 (70.6 %) samples were identified as multiple-clone infections, for which at least one locus contained more than one allele. The greatest number of multiple-clone infections was identified using *msp1B10* and the microsatellite markers (27 and 24 % of all samples, respectively) (Fig. 3a). Conversely, the *msp1B2* marker only identified three (5.9 %) multiple-clone infections. A detailed analysis of the data showed that three additional infections were likely lost by the *msp1B2* marker due to a weak fluorescent signal for the rare allele. Moreover, half of the 36 multi-clonal infections defined using the one-fourth criterion were identified using two or more markers (Fig. 3b). *msp1B10* identified 38 % of the multiple-clone infections, and this proportion increased to 68 % when *msp1B10* was combined with *PvMS6* (Fig. 3b). The further addition of *PvMS7* and *MN21* detected 95 % of the clonal infections. Combined with an analysis based on *msp3α*, the percentage of identified multi-clonal infections reached 100 %.

The potential of each marker to identify polymorphisms can vary considerably and reflects an estimate of the multiplicity of infection. For this part of the study, many samples (22 out 51) that were selected have been previously characterized as multiple-clone infections [9, 11, 34]. Thus, they were not used to determine the rate of multi-clonal infection and the genetic diversity of this parasite population in order to prevent overestimation. To compare the genetic diversity of parasite populations estimated by these markers, the available data for *P. vivax* from different geographic regions were compiled. The estimates of diversity were highest for the antigen-encoding loci *msp1B10* (*H*_E_ range 0.880–0.902) and *msp1B2* (0.813–0.875) (Additional file 6). For the microsatellites *PvMS6,PvMS7*, and *MN21*, only data from Brazil were available and the estimate of diversity was lower (mean *H*_E_ of 0.584, 0.785 and 0.650, respectively).

## Discussion

Numerous studies have revealed substantial clonal diversity of *Plasmodium* within its vertebrate hosts. The characterization of this diversity can influence treatment outcome and elucidate within-host dynamics that may be shaped by several factors, such as host immunity, density-dependent control mechanisms and drug treatment [12, 16, 31]. Furthermore, estimates of within-host variability are relevant to correctly infer evolutionary and population genetics parameters, e.g., selection and recombination [13, 14]. To infer the multiplicity of infection of *P. vivax*, a panel of suitable molecular markers was defined herein, which included microsatellites, a tandem repeat and antigen-coding genes.

The analysis of artificial DNA mixtures with well-defined proportions of cloned products showed that the commonly used *msp1* antigen-coding marker and the tandem repeat *MN21* allowed for the estimation of the expected ratio of both alleles in the majority of preparations when using normalized data. Conversely, the microsatellite markers were sensitive to the decreased relative abundance of alleles but, in addition to *msp3α,* did not accurately estimate the relative clonal proportions in artificial mixtures. For example, a preferential amplification of the shortest allele (with fewest repeats) for *PvMS6* was consistently observed in tested dilutions. Accordingly, the preferential amplification of alleles of differing length was previously reported for other microsatellite and antigen-coding loci [32–34]. As indicated by Walsh et al. [27], the extent of preferential amplification is related to the size difference between the allelic PCR products, which was significantly greater for *PvMS6* (102 bp). Although not assessed in this study, several other conditions may lead to preferential PCR amplification, such as significant differences in the GC content between alleles, stochastic fluctuation in the presence of low amounts of target DNA molecules [33], and reduced amplification efficiency due to sequence polymorphism in the primer-binding site [35]. Finally, the capillary-based instrument itself may introduce errors in measured relative density of the PCR product for each allele. However, instrument-based errors are unlikely in this study because the results presented here indicate that the method is reproducible. Specifically, replicate experiments yielded similar results, as also reported in other studies [24].

Although some markers, such as the antigen-coding marker *msp1* and the tandem repeat, provide an indication of the actual relative proportions of clones in artificial infections, the quantification yielded by the method described herein is subjected to limitations. First, traditional end-point PCR may lead to bias in the template-to-product ratios of target sequences amplified during PCR, particularly for nested PCR, due to increasing numbers of PCR cycles [36, 37]. Thus, the amplification bias observed in the 1:1 mixtures and other dilutions of microsatellites and *msp3α* may be the result of reaction saturation (plateau phase). At later cycles, the efficiency of PCR eventually declines for a number of reasons, including the exhaustion of reagents and the enzyme, the accumulation of inhibitors and the rehybridization of PCR products, which may interfere with primer binding or extension [37–39]. As a consequence, templates that reach inhibitory concentrations essentially stop amplifying while others continue to efficiently undergo amplification. In addition, the results shown here and by others indicate that accurate quantification requires normalization using a baseline mixture of known proportion to calibrate samples to be analysed [19, 24, 25]. Such normalization restricts the use of this method for experimental infection models. To circumvent these limitations, quantitative PCR (qPCR) or next-generation sequencing (NGS) may be applied to more reliably estimate the relative abundance of clones in an infection. Whereas qPCR is restricted to known single-species infections, like the method described here [34], the applications of NGS are much broader. As recently published for *P. falciparum* and *P. vivax*, NGS robustly represents clonal multiplicity and is very promising for drug-resistance or population genetics studies [40–43]. Nevertheless, genomic-level studies remain infeasible in many settings.

Many criteria have been used to differentiate rare alleles from artifacts, such as stutter (peaks that result from DNA strand slippage during PCR at intervals corresponding to nucleotide repeat sizes) or non-specific peaks [11, 11, 26]. Because the sensitivity and specificity of these criteria can vary, comparing studies that applied different criteria is problematic. The present study sought to compare the rates at which multi-clonal infections were detected using the two criteria that are frequently applied to score multiple alleles per locus, i.e., minor peaks ≥33 % or ≥25 % of the height of the predominant peak. Overall, the one-fourth criterion allowed the detection of rare alleles in more unbalanced mixtures and the detection of significantly more alleles, especially for microsatellite markers. The results clearly show that when the one-third criterion is applied for microsatellites, multi-clonal infections may be underestimated, even when the clones were present in similar proportions. This finding was not surprising because several technical difficulties have been described related to the scoring of microsatellite alleles, such as the preferential amplification of alleles with fewer repeats and the higher probability of failing to detect the less abundant alleles [19, 25].

This study examined six loci were of differing molecular features that have been widely used in studies of population genetics and the molecular epidemiology of vivax malaria [9, 12, 11, 34]. These loci included selectively neutral and non-neutral markers on different chromosomes and markers differing in mutation rate (microsatellites, a tandem repeat and an antigen-encoding gene). Thus, most of the variability of *P. vivax* populations should have been captured, and the within-host diversity should have been effectively characterized in different epidemiological settings. In these areas, several factors may modulate the genetic variability of a locus and/or of complete genomes, such as the malaria transmission rates, selective constraints imposed by the host’s immunity and anti-malarial drug use, and the historical-demographic processes of the parasite population. Compiling the available data on the genetic diversity of *P. vivax* from different geographic regions higher estimates of diversity were found for antigen-encoding loci and less variable estimates for microsatellites and tandem repeat loci. When samples from the Brazilian Amazon were genotyped for all six markers, *msp1B10*, microsatellite markers and the tandem repeat *MN21* yielded similar results. Interestingly, a significant difference was observed between blocks 2 and 10 of *msp1*. Whereas *msp1B10* was able to detect almost 40 % of the 36 multi-clonal infections identified, *msp1B2* allowed the detection of only 6 % of these infections. Moreover, *msp3α* also showed poor performance in the detection of multiple-clone infections from field samples. Because these loci encode antigens exposed to the immune system, these results suggest that the patterns observed could reflect the different regimes of immune selection that these antigens are exposed to in this population. Notably, 50 % of the multiple-clone infections were detected by only one marker, indicating that adding additional loci may further increase the probability of detecting these infections.

Determining whether a sample is mono-clonal or contains multiple clones of the parasite requires the careful selection of markers because most randomly selected markers may not faithfully depict the true complexity of an infection. Based on the performances of the six markers used here to characterize parasite diversity in both artificial mixtures of clones and in field samples, the combination, including *PvMS6*, *PvMS7*, *MN21*, and *msp1B10* were selected for use in molecular epidemiology studies. These markers comprise a panel containing highly informative loci that exhibit genetic variability in all examined *P. vivax* populations as well as loci that are less sensitive to PCR amplification biases and were able to better characterize the multiplicity of infection. In this study, a PCR-CE was proposed for allele scoring, which has the advantage of allowing the accurate and fast genotyping of a large number of field samples at relatively low cost. Furthermore, a less stringent criterion for rare allele identification significantly increases the sensitivity of this method. Thus, a minimum of one-fourth should be used as a cut-off value for minor peak detection. Although the results described apply to a small subset of alleles and field samples likely contain several unknown alleles, they reveal information about amplification bias, which can be used to identify markers and conditions under which such biases are minimized. Field samples from the Brazilian Amazon region, an area of low and unstable malaria transmission, were used to define the proposed panel. Nevertheless, further characterization is required, especially in regions of high endemicity, where this panel would also provide information about multi-clonal infections due to the high genetic variability of parasite population in such areas.

## Conclusions

This study showed that the analysed molecular markers varied in their ability to detect and estimate the relative abundance of different clones in a *P. vivax* infection, which may bias the estimated of the genetic diversity of the parasite population. Using artificial mixtures of DNA and the genotyping of field isolates from Brazil, four markers (two microsatellites, one tandem repeat and one antigen-encoding gene) were selected based on their ability to characterize the within-host parasite diversity. Moreover, the use of a less stringent criterion is recommended to increase the probability of detecting rare alleles.

## Additional files

**Additional file 1.** Allele frequencies (%) and genetic diversity analysis of molecular markers of *Plasmodium vivax* isolates from Brazil.
**Additional file 2.** Expected and observed ratio of alleles amplified from 11 artificial mixtures of plasmid DNA. The relative abundance of alleles was estimated as the ratio between the heights of the peaks (measured in arbitrary fluorescence units).
**Additional file 3.** Relative abundance of alleles of the six molecular markers in the mixtures of plasmid DNA. The relative abundance of alleles was estimated as the ratio between the heights of the peaks for non-normalized data: A-type allele (in grey) and B-type allele (black).
**Additional file 4.** Relative abundance of *Plasmodium vivax* molecular marker alleles amplified from mixtures of genomic DNA samples from two *Plasmodium vivax*-infected patients.
**Additional file 5.** Ratio of rare to predominant alleles in the artificial mixtures of plasmid DNA.
**Additional file 6.** Summary of previous studies reporting the genetic diversity and multiplicity of infection of *Plasmodium vivax*.

## Abbreviations

*msp1B2*: block 2 of *merozoite surface protein 1* gene
*msp1B10*: block 10 of *merozoite surface protein 1* gene
*msp3α*: *merozoite surface protein 3 alpha* gene
PCR-CE: PCR-capillary electrophoresis-based method
PCR-RFLP: PCR-Restriction fragment length polymorphism
pDNA: DNA plasmid
gDNA: genomic DNA
*H*_E_: virtual expected heterozygosity
SNP: single nucleotide polymorphism
rFU: arbitrary fluorescence units
