# Supplementary material for: Multiple-clone infections of Plasmodium vivax: definition of a panel of markers for molecular epidemiology
Source: Malar J. 2015 Aug 25;14:330. doi: 10.1186/s12936-015-0846-5 (PMC4548710; doi:10.1186/s12936-015-0846-5)
Supplement: Supplementary file 1 — Additional file 1. Allele frequencies (%) and genetic diversity analysis of molecular markers of Plasmodium vivax isolates from Brazil. [file 12936_2015_846_MOESM1_ESM.docx]

**Additional file 1**. Allele frequencies (%) and genetic diversity analysis of molecular markers of *Plasmodium vivax* isolates from Brazil.

^a^ Fragment size in base pairs
