# Supplementary material for: Multiple-clone infections of Plasmodium vivax: definition of a panel of markers for molecular epidemiology
Source: Malar J. 2015 Aug 25;14:330. doi: 10.1186/s12936-015-0846-5 (PMC4548710; doi:10.1186/s12936-015-0846-5)
Supplement: Supplementary file 2 — Additional file 2. Expected and observed ratio of alleles amplified from 11 artificial mixtures of plasmid DNA. The relative abundance of alleles was estimated as the ratio between the heights of the peaks (measured in arbitrary fluorescence units). [file 12936_2015_846_MOESM2_ESM.docx]

**Additional file 2**. Expected and observed ratio of alleles amplified from 11 artificial mixtures of plasmid DNA. The relative abundance of alleles was estimated as the ratio between the heights of the peaks (measured in arbitrary fluorescence units). Data represent the average of two independent experiments.

^a^Expected ratios refer to the proportion of molecules (cloned DNA) from each allele used as a template for PCR amplification. The fragments cloned into pGEM-T Vector and TOPO Cloning Vector were called A-type and B-type, respectively.

^b^The observed data were normalized to facilitate data interpretation.

^c^We have highlighted (in bold and underlined) the data that are not significantly different from their expected ratios (Fisher´s exact test, *P* > 0.05).The relationship between expected and normalized observed ratios of peak heights was modeled by regression to determine the linear fit of the data in the mixtures with an excess of A-type allele (A): *PvMS6* (*R^2^* = 95.00, slope = 1.679, *P* = 0.001), *PvMS7* (*R^2^* = 99.30, slope = 0.506, *P* = 0.000), *MN21* (*R^2^* = 97.31, slope = 1.389, *P* = 0.000), *msp3α* (*R^2^* = 87.61, slope = 0.214, *P* = 0.006), *msp1B2* (*R^2^* = 99.27, slope = 1.273, *P* = 0.000) and *msp1B10* (*R^2^* = 99.32, slope = 1.040, *P* = 0.000). Relationship between expected and normalized observed data for mixtures with predominance of B-type allele (B): *PvMS6* (*R^2^* = 81.65, slope = 0.417, *P* = 0.013), *PvMS7* (*R^2^* = 82.16, slope = 0.458, *P* = 0.013), *MN21* (*R^2^* = 98.24, slope = 0.709, *P* = 0.000), *msp3α* (*R^2^* = 75.07, slope = 0.435, *P* = 0.026), *msp1B2* (*R^2^* = 97.94, slope = 1.089, *P* = 0.000), *msp1B10* (*R^2^* = 98.27, slope = 1.303, *P* = 0.000).
