# Supplementary figures and images for: Multiple-clone infections of Plasmodium vivax: definition of a panel of markers for molecular epidemiology
Source: Malar J. 2015 Aug 25;14:330. doi: 10.1186/s12936-015-0846-5 (PMC4548710; doi:10.1186/s12936-015-0846-5)

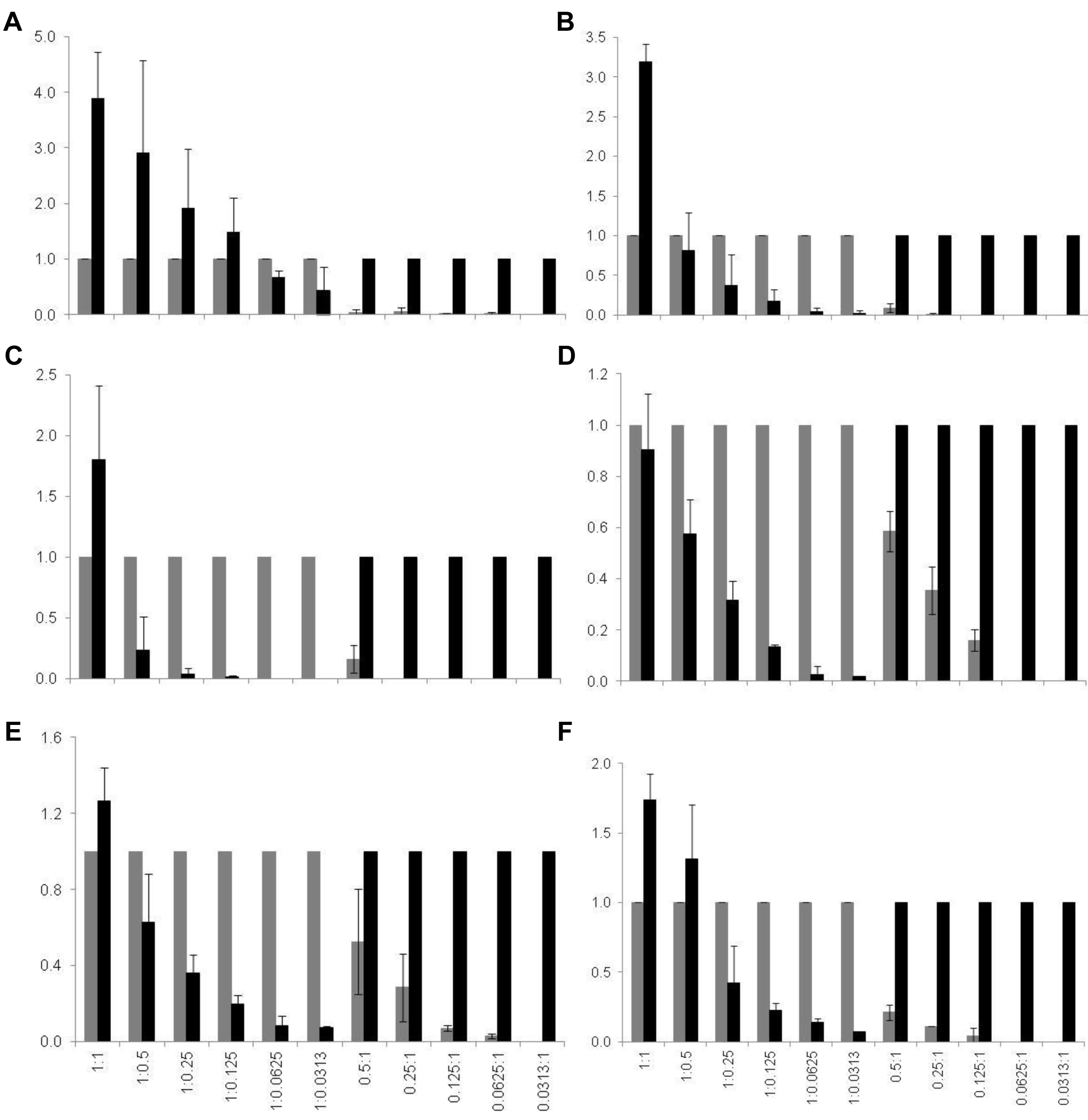

Supplement: Supplementary file 3 — Additional file 3. Relative abundance of alleles of the six molecular markers in the mixtures of plasmid DNA. The relative abundance of alleles was estimated as the ratio between the heights of the peaks for non-normalized data: A-type allele (in grey) and B-type allele (black). [file 12936_2015_846_MOESM3_ESM.tif]
