# Supplementary material for: Multiple-clone infections of Plasmodium vivax: definition of a panel of markers for molecular epidemiology
Source: Malar J. 2015 Aug 25;14:330. doi: 10.1186/s12936-015-0846-5 (PMC4548710; doi:10.1186/s12936-015-0846-5)
Supplement: Supplementary file 4 — Additional file 4. Relative abundance of Plasmodium vivax molecular marker alleles amplified from mixtures of genomic DNA samples from two Plasmodium vivax-infected patients. [file 12936_2015_846_MOESM4_ESM.docx]

**Additional file 4**. Relative abundance of *P. vivax* molecular marker alleles amplified from mixtures of genomic DNA samples from two *P. vivax*–infected patients.

*Normalized data that are not significantly different from their expected ratios using Fisher´s exact test (*P* > 0.05) are highlighted in bold and underlined.
