# Supplementary material for: Multiple-clone infections of Plasmodium vivax: definition of a panel of markers for molecular epidemiology
Source: Malar J. 2015 Aug 25;14:330. doi: 10.1186/s12936-015-0846-5 (PMC4548710; doi:10.1186/s12936-015-0846-5)
Supplement: Supplementary file 5 — Additional file 5. Ratio of rare to predominant alleles in the artificial mixtures of plasmid DNA. [file 12936_2015_846_MOESM5_ESM.docx]

**Additional file 5**. Ratio of rare to predominant alleles in the artificial mixtures of plasmid DNA.

^a^Expected ratios refer to the proportion of molecules (cloned DNA) from each allele used as a template for PCR amplification.

^b^The relative abundance of alleles was estimated as the ratio between the heights of the peaks (measured in arbitrary fluorescence units) without normalization.

^c^Values above 25% are underlined, while those that fit only the one-third criterion (≥33%) are highlighted in bold.
