# Supplementary material for: Multiple-clone infections of Plasmodium vivax: definition of a panel of markers for molecular epidemiology
Source: Malar J. 2015 Aug 25;14:330. doi: 10.1186/s12936-015-0846-5 (PMC4548710; doi:10.1186/s12936-015-0846-5)
Supplement: Supplementary file 6 — Additional file 6. Summary of previous studies reporting the genetic diversity and multiplicity of infection of Plasmodium vivax. [file 12936_2015_846_MOESM6_ESM.docx]

**Additional file 6**. Summary of previous studies reporting the genetic diversity and multiplicity of infection of *Plasmodium vivax*.

| Genetic marker | Nº Sample | Region | *H_E_*^a^ | Nº Allele | Multi-clone infections rate (%) ^b^ | Nº markers^c^ | Methodology | Reference |
| --- | --- | --- | --- | --- | --- | --- | --- | --- |
| *PvMS6* | 53 | Brazil | 0.521 | 19 | 0.57 | 11 | PCR-CE | [1] |
|  | 65 |  | 0.647 | 12 | - | - | PCR-CE | [2] |
| *PvMS7* | 44 | Brazil | 0.782 | 11 | 0.57 | 11 | PCR-CE | [1] |
|  | 65 |  | 0.789 | 8 | - | - | PCR-CE | [2] |
| *MN21* | 44 | Brazil | 0.650 | 6 | ≈0.46 | 5 | PCR-gel based electrophoresis^d^ | [3] |
| *msp1B2* | 65 | Brazil | 0.813 | 7 | - | - | PCR-CE | [2] |
|  | 28 |  | - | 7 | - | - | Sequencing | [4] |
|  | 100 | Thailand | - | 5 | 0.26 | 4 | PCR-RFLP | [5] |
|  | 18 |  | - | 11 | - | - | Sequencing |  |
|  | 100 | Papua New  Guinea (PNG) | 0.875 | 25 | ­- | - | PCR-CE | [6] |
| *msp1B10* | 65 | Brazil | 0.886 | 12 | - | - | PCR-CE | [2] |
|  | 100 | Thailand | ­- | 4 | 0.26 | 4 | PCR-RFLP | [5] |
|  | 8 |  | - | 6 | - | - | Sequencing |  |
|  | 108 | PNG | 0.902 | 28 | ­- | - | PCR-CE | [6] |
|  | 1,094 |  | 0.881 | 57 | 0.74 | 2 | PCR-CE | [7] |
|  | 1,120 |  | 0.880 | ­- | ­- | - | PCR-CE | [8] |
| *msp3α* | 52 | Brazil | 0.270 | 11 | ­- | - | PCR-RFLP | [9] |
|  | 55 | Colombia | - | 12 | 0.36 | 1 | PCR-RFLP | [10] |
|  | 27 | Venezuela | - | 9 | - | - | Sequencing | [11] |
|  | 10 |  | - | 4 | - | - | Sequencing | [12] |
|  | 109 | French Guiana | ­- | 11 | 0.21 | 2 | PCR-RFLP | [13] |
|  | 88 | Thailand | ­- | 13 | 0.36 | 2 | PCR-RFLP | [14] |
|  | 17 |  | - | 13 | - | - | Sequencing | [12, 15] |
|  | 16 | PNG | ­- | 11 | 0.23 | 1 | PCR-RFLP | [16] |
|  | 95 |  | ­- | 24 | - | - | PCR-RFLP | [17] |
|  | 106 |  | 0.806 | 15 | ­- | - | PCR-CE | [6] |
|  | 28 | Republic of Korea | ­- | 5 | 0 | 1 | Sequencing | [18] |
|  | 151 | India | - | 37 | 0.11 | 3 | PCR-RFLP | [19] |
|  | 22 |  | - | 21 | - | - | PCR-RFLP | [20] |
|  | 130 | Pakistan | - | 23 | 0.05 | 2 | PCR-RFLP | [21] |
|  | 50 | Iran (North) | - | 12 | 0.04 | 1 | PCR-RFLP | [22] |
|  | 94 | Iran (South) | - | 49 |  |  |  |  |
|  | 337 | Pakistan and Iran | ­- | 16 | ≈0.27 | 3 | PCR-RFLP | [23] |

^a^ Virtual expected heterozygosity, i.e., the probability that a pair of alleles randomly selected from the population are different.

^b^ Percentage of positive samples that carry multiple clones of *P. vivax*.

^c^ Number of markers used to estimate multiple-clone infection rates.

^d^ The PCR product was separated and analysed in a gel-based DNA sequencer.

References

1. Rezende A, Tarazona-Santos E, Fontes C, Souza J, Couto A, Carvalho L, et al. **Microsatellite loci: determining the genetic variability of *Plasmodium vivax***. *Trop Med Int Health*. 2010;**15**:718–26.

2. de Araujo FC, de Rezende AM, Fontes CJ, Carvalho LH, de Brito CFA. **Multiple-clone activation of hypnozoites is the leading cause of relapse in *Plasmodium vivax* infection**. *PLoS One*. 2012;**7**:e49871.

3. Rezende AM, Tarazona-Santos E, Couto AD, Fontes CJ, De Souza JM, Carvalho LH, et al. **Analysis of genetic variability of *Plasmodium viva*x isolates from different Brazilian Amazon areas using tandem repeats**. *Am J Trop Med Hyg*. 2009;**80**:729–33.

4. Soares LA, Evangelista J, Orlandi PP, Almeida ME, de Sousa LP, Chaves Y, et al. **Genetic diversity of MSP1 Block 2 of *Plasmodium vivax* isolates from Manaus (central Brazilian Amazon)**. *J Immunol Res*. 2014;**2014**:671050.

5. Imwong M, Pukrittayakamee S, Grüner A, Rénia L, Letourneur F, Looareesuwan S, et al. **Practical PCR genotyping protocols for *Plasmodium vivax* using Pvcs and Pvmsp1**. *Malar J*. 2005;**4**:20.

6. Koepfli C, Mueller I, Marfurt J, Goroti M, Sie A, Oa O, et al. **Evaluation of *Plasmodium vivax* genotyping markers for molecular monitoring in clinical trials**. *J Infect Dis*. 2009;**199**:1074–80.

7. Koepfli C, Ross A, Kiniboro B, Smith TA, Zimmerman PA, Siba P, et al. **Multiplicity and diversity of *Plasmodium vivax* infections in a highly endemic region in Papua New Guinea**. *PLoS Negl Trop Dis*. 2011;**5**:e1424.

8. Koepfli C, Schoepflin S, Bretscher M, Lin E, Kiniboro B, Zimmerman PA, et al. **How much remains undetected? Probability of molecular detection of human Plasmodia in the field**. *PLoS One*. 2011;**6**:e19010.

9. Ribeiro RS, Ladeira L, Rezende AM, Fontes CJ, Carvalho LH, Brito CF. **Analysis of the genetic variability of PvMSP-3alpha among *Plasmodium vivax* in Brazilian field isolates**. *Mem Inst Oswaldo Cruz*. 2011;**106**(Suppl 1):27–33.

10. Cristiano FA, Perez MA, Nicholls RS, Guerra AP. **Polymorphism in the *Plasmodium vivax* msp 3: gene in field samples from Tierralta, Colombia**. *Mem Inst Oswaldo Cruz*. 2008;**103**:493–6.

11. Ord R, Polley S, Tami A, Sutherland CJ. **High sequence diversity and evidence of balancing selection in the Pvmsp3alpha gene of *Plasmodium vivax* in the Venezuelan Amazon**. *Mol Biochem Parasitol*. 2005;**144**:86–93.

12. Rice BL, Acosta MM, Pacheco MA, Escalante AA. **Merozoite surface protein-3 alpha as a genetic marker for epidemiologic studies in *Plasmodium vivax*: a cautionary note**. *Malar J*. 2013;**12**:288.

13. Veron V, Legrand E, Yrinesi J, Volney B, Simon S, Carme B. **Genetic diversity of msp3alpha and msp1_b5 markers of *Plasmodium vivax* in French Guiana**. *Malar J*. 2009;**8**:40.

14. Cui L, Mascorro C, Fan Q, Rzomp K, Khuntirat B, Zhou G, et al. **Genetic diversity and multiple infections of *Plasmodium vivax* malaria in Western Thailand**. *Am J Trop Med Hyg*. 2003;**68**:613–9.

15. Mascorro CN, Zhao K, Khuntirat B, Sattabongkot J, Yan G, Escalante AA, et al. **Molecular evolution and intragenic recombination of the merozoite surface protein MSP-3alpha from the malaria parasite *Plasmodium vivax* in Thailand**. *Parasitology*. 2005;**131**:25–35.

16. Bruce MC, Galinski MR, Barnwell JW, Snounou G, Day KP. **Polymorphism at the merozoite surface protein-3alpha locus of *Plasmodium vivax*: global and local diversity**. *Am J Trop Med Hyg*. 1999;**61**:518–25.

17. Bruce MC, Galinski MR, Barnwell JW, Donnelly CA, Walmsley M, Alpers MP, et al. **Genetic diversity and dynamics of *Plasmodium falciparum* and *P. vivax* populations in multiply infected children with asymptomatic malaria infections in Papua New Guinea**. *Parasitology*. 2000;**121**(Pt 3):257–72.

18. Han ET, Song TE, Park JH, Shin EH, Guk SM, Kim TY, et al. **Allelic dimorphism in the merozoite surface protein-3alpha in Korean isolates of *Plasmodium vivax***. *Am J Trop Med Hyg*. 2004;**71**:745–9.

19. Kim J, Imwong M, Nandy A, Chotivanich K, Nontprasert A, Tonomsing N, et al. **Genetic diversity of *Plasmodium vivax* in Kolkata, India**. *Malar J*. 2006;**5**:71.

20. Prajapati SK, Joshi H, Valecha N. ***Plasmodium vivax* merozoite surface protein-3 alpha: a high-resolution marker for genetic diversity studies**. *J Vector Borne Dis*. 2010;**47**:85–90.

21. Khan SN, Khan A, Khan S, Ayaz S, Attaullah S, Khan J, et al. **PCR/RFLP-based analysis of genetically distinct *Plasmodium vivax* population of Pvmsp-3alpha and Pvmsp-3beta genes in Pakistan**. *Malar J*. 2014;**13**:355.

22. Zakeri S, Barjesteh H, Djadid ND. **Merozoite surface protein-3alpha is a reliable marker for population genetic analysis of *Plasmodium vivax***. *Malar J*. 2006;**5**:53.

23. Zakeri S, Raeisi A, Afsharpad M, Kakar Q, Ghasemi F, Atta H, et al. **Molecular characterization of *Plasmodium vivax* clinical isolates in Pakistan and Iran using pvmsp-1, pvmsp-3alpha and pvcsp genes as molecular markers**. *Parasitol Int*. 2010; **59**:15-21.
